# Efficacy and Safety of Second and Third-Generation Laser Balloon for Paroxysmal Atrial Fibrillation Ablation Compared to Radiofrequency Ablation: A Matched-Cohort

**DOI:** 10.3390/jcdd8120183

**Published:** 2021-12-13

**Authors:** Charles Guenancia, Nefissa Hammache, Clémence Docq, Karim Benali, Darren Hooks, Mathieu Echivard, Nathalie Pace, Isabelle Magnin-Poull, Christian de Chillou, Jean-Marc Sellal

**Affiliations:** 1 Département de Cardiologie, CHRU de Nancy, 54500 Vandœuvre-lès-Nancy, France; n.hammache@chru-nancy.fr (N.H.); clemence.docq@laposte.net (C.D.); k.benali@chru-nancy.fr (K.B.); m.echivard@chru-nancy.fr (M.E.); n.pace@chru-nancy.fr (N.P.); i.magnin@chru-nancy.fr (I.M.-P.); c.dechillou@chru-nancy.fr (C.d.C.); jm.sellal@chru-nancy.fr (J.-M.S.); 2 INSERM-IADI, U1254, 54500 Vandœuvre lès-Nancy, France; 3 Département de Cardiologie, CHU de Dijon, 21000 Dijon, France; 4 Département de Cardiologie, CHU de Saint-Etienne, 42270 Saint-Priest-en-Jarez, France; 5 Cardiology Department, Wellington Hospital, Wellington 6021, New Zealand; darren.hooks@ccdhb.org.nz

**Keywords:** atrial fibrillation, ablation, laser, radiofrequency, paroxysmal

## Abstract

Laser balloon (LB) has emerged as an interesting strategy for pulmonary vein isolation in paroxysmal atrial fibrillation (AF). A third-generation LB has recently been developed, allowing a continuous ablation set. We aimed to compare the results from our center’s experience with second and third-generation LBs to a cohort of matched patients who had undergone radiofrequency ablation (RFA) with contact-force catheters. This retrospective monocenter case-control study included our first 50 LB paroxysmal AF ablations (26 second and 24 third-generation LB) and 50 RFA controls, matched on age, sex and left atrial dilation. The two groups had similar baseline parameters. LB procedures were significantly shorter than RFA (129 (110–160) vs. 160 (119–198) min, *p* = 0.007). During AF ablation, two major complications occurred in each group. At the one-year follow-up, AF recurrence was diagnosed in 7 (14%) of the LB group vs. 14 (28%) of the RFA group (*p* = 0.14). Moreover, we observed that third-generation LB procedures were associated with shorter laser applications (22 (19–29) vs. 69 (55–76) min, *p* < 0.001) and procedural durations (111 (100–128) vs. 151.5 (128.5–167) min, *p* < 0.001) compared to second-generation LB procedures. In the context of the major increase in the number of AF ablations, LB demonstrated consistent results in terms of clinical success, complications and also reduced procedure durations compared to RFA.

## 1. Introduction

Paroxysmal atrial fibrillation (AF) ablation has become first-line therapy in symptomatic patients with drug-refractory AF, and more recently, an alternative to antiarrhythmic therapy depending on patient choice [1]. In paroxysmal AF patients, pulmonary vein isolation (PVI) is the cornerstone of ablation procedures [2,3]. PVI has been widely demonstrated as highly effective at reducing clinical recurrences of AF. PVI can be achieved using a variety of techniques, including radiofrequency ablation (RFA), cryoballoon ablation (CB), and more recently, laser balloon (LB) ablation [4].

The LB technology (HeartLight, CardioFocus, Marlborough, MA, USA) allows direct visualization of the pulmonary veins through an endoscopic view, and thanks to the compliance of the balloon, it can be adapted to all venous anatomies. Moreover, it has been suggested that LB ablation would result in reduced endothelial lesions and atrial wall edema [5,6]. The first-generation LB demonstrated non-inferior safety and efficacy for treatment of paroxysmal atrial fibrillation (PAF) compared to RFA [7], but was associated with longer procedure durations. A randomized study also demonstrated similar results for the second-generation LB (Excalibur) compared to RFA in persistent AF ablation [8]. These first two generations were based on point-by-point energy delivery. Each ablation point (20–30 s duration) had to be manually positioned with a 25–30% overlap with the previous point in order to provide continuous circumferential ablation around the PV ostium. More recently, a third-generation LB (X3) was developed, including a new ablation mode (RAPID). This mode provides automatic movement of laser delivery using a motor included in the handle of the catheters. Continuous circular energy delivery is achieved in 180 s. A large single-center randomized trial demonstrated the non-inferiority of this third-generation LB compared to cryotherapy [9]. This technical innovation resulted in a higher PVI durability in patients with recurrent AF compared to the second-generation LB [10]. In the meantime, RFA has also benefited from major improvements, including the implementation of contact-force (CF) sensors at the tip of ablation catheters and the development of dedicated ablations indices to improve the reproducibility and safety of AF ablations procedures [11,12].

We, therefore, aimed to compare the results from our center’s experience with second and third-generation LB paroxysmal AF ablation to a cohort of matched patients who had undergone RFA using CF catheters in terms of clinical success at one year, but also on the complication rate and procedural characteristics.

## 2. Materials and Methods

### 2.1. Study Population

This retrospective case-control observational study included the first 50 consecutive adult patients with symptomatic drug-refractory paroxysmal AF referred for a first ablation using either second-generation (Excalibur) or third-generation (X3) LB in Nancy University Hospital from July 2019 to September 2020.

For the RFA-matched cohort, a total of 417 patients who had a first RF paroxysmal AF ablation between April 2015 and December 2018 at Nancy University Hospital and a one-year follow-up were screened from a previous database [13]. A 1:1 case-control matching with LB patients was then performed (on age, sex and indexed left atrial volume).

AF was considered to be paroxysmal if it terminated spontaneously or with intervention within 7 days of onset.

Inclusion criteria were: first procedure of catheter ablation for PAF and 12-month follow-up. Exclusion criteria were: prior AF catheter ablation and CB ablation, left atrial (LA) linear lesions or LA defragmentation. All patients were adults and provided written informed consent for the AF ablation, and all procedures were in line with current guidelines.

The medical software DxCare^®^ was used to collect all patients’ data needed for the study. We evaluated: demographic and physical features, comorbidities, treatments, electrocardiogram features at the beginning of the procedure and at hospital discharge and ablation procedure modalities. Renal failure was defined as a glomerular filtration rate (MDRD) < 60 mL/min/1.73m^2^.

### 2.2. Echocardiography

Transthoracic and transesophageal echocardiography were performed within 24 h before intervention, using a Vivid S6 cardiovascular ultrasound system (General Electric, Horten, Norway). The following data were collected: left ventricular ejection fraction (biplane Simpson’s method), LA surface area (apical four-chamber view at end-systole), indexed LA volume (biplane area-length method at end-systole) and LA diameter (parasternal long-axis view). LA dilation was defined as indexed LA volume 34 mL/m^2^ [14].

### 2.3. Ablation Procedure

All ablations were performed under local anesthesia and conscious sedation using intravenous nalbuphine and/or midazolam. After femoral venous access, the transseptal puncture was performed using an 8F sheath and a Brockenbrough needle with fluoroscopy guidance. Intravenous heparin was administered as boluses and as a continuous infusion to maintain an activated clotting time ≥ 300 s.

#### 2.3.1. LB Ablation

All procedures were performed by a single senior operator (JMS). The transseptal sheath was exchanged for the 12F deflectable sheath. Preablation electrical mapping of pulmonary veins (PV) potentials was performed using a circular mapping catheter (Lasso, Biosense Webster, Diamond Bar, CA, USA). No electroanatomical mapping system was used during LB procedures.

**Figure 1:**
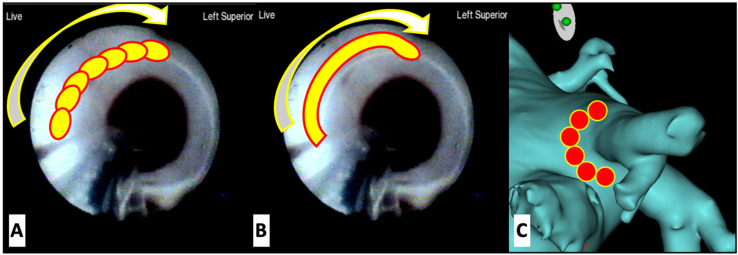
(**A**,**B**): Endoscopic view of the left superior pulmonary vein with schematic representation of laser administration. Energy is delivered sequentially with Excalibur generation laser balloons (**A**) and continuously with X3 generation laser balloons (**B**). (**C**) Representation of the radiofrequency isolation of the pulmonary vein.

As previously described [15], using the deflectable sheath, the Excalibur (second-generation) or the X3 (third-generation) LB was positioned at the ostium of the target PV, and the balloon was inflated (Figure 1). Ablation was performed under visual guidance. For Excalibur procedures, ablation consisted of the deployment of laser energy in a point-by-point manner, overlapping each lesion by 30–50% and for X3 procedures of either RAPID mode or manual mode energy delivery or a combination of both. After placement of the initial anatomically guided encircling lesion set, the circular mapping catheter was used to assess for electrical isolation of the PV. If the PV was not isolated, LB was again used to deliver lesions to the area of electrical breakthrough or alternatively, another lesion set completely encircling the PV was delivered. During ablation of the right-sided PVs, phrenic nerve pacing was always performed from the superior vena cava to minimize the risk of phrenic nerve injury by monitoring for diaphragmatic movement.

After 15 min post-ablation, PVs were reassessed for electrical isolation. A circular mapping catheter was used to identify the entrance block.

#### 2.3.2. RFA

All procedures were performed by one of the three senior operators of the center (JMS, IMP, CDC). Two catheters were advanced from the right femoral vein to the LA through the transseptal puncture, under fluoroscopic guidance: a 10-pole circular mapping catheter (Lasso, Biosense Webster, Diamond Bar, CA, USA or Inquiry AFocus II, St. Jude Medical, St. Paul, MN) and a 3.5-mm externally irrigated-tip ablation catheter. A steerable quadripolar catheter (Xtrem, SORIN Group, Clamart, France) was placed into the coronary sinus and used as an electroanatomical mapping reference.

A three-dimensional navigation system (CARTO^®^, Biosense Webster, Inc., Irvine, CA, USA or EnSite NavX system St. Jude Medical, St Paul, MN, USA) was used to create a three-dimensional electroanatomical map of the LA, which was integrated with computed tomography of the LA. PVI was performed with radiofrequency energy in a point-by-point wide area circumferential ablation (two by two PVI) pattern using a Thermocool SmartTouch irrigated tip CF-sensing ablation catheter (Biosense Webster, Inc., Irvine, CA, USA) or a Tacticath SE^TM^ Ablation Catheter (St. Jude Medical, St. Paul, MN, USA) introduced via a non-steerable sheath. Each lesion was respectively guided by ablation index (AI) targets using 450 at the roof and anterior walls, and 350 at the posterior and inferior walls or lesion size index (LSI) targets using 5.5 at the roof and anterior walls and 4.5 at the posterior and inferior walls. RF pulses were delivered by using a 550-kHz RF Stockert-Cordis generator and the ablation catheter, in a power-controlled mode, with RF energy up to 30 Watts at the anterior part of the veins and 25 Watts at their posterior part. After 15 min post-ablation, PVs were reassessed for electrical isolation. A circular mapping catheter was used to identify the entrance block.

### 2.4. Patient Follow-Up

All patients underwent an electrocardiographic evaluation before being discharged from hospital. AF recurrences were assessed after a 3-month blanking period and defined as an ≥1 AF episode recorded during a 12-lead ECG or ≥1 AF episode lasting ≥ 30 s documented by Holter monitoring. Arrhythmia monitoring included clinical evaluation, 12-lead electrocardiogram in case of symptom recurrence, and systematic 24-h Holter monitor recording by the referring cardiologist at months 3, 6 and 12. Our investigative team was unaware of the follow-up assessment outcomes.

The continuation or initiation of antiarrhythmic drug therapy post-procedure and at 3 months was left to the referring physician preference. Successful ablation was defined as the absence of a documented AF episode with or without antiarrhythmic at one year after the procedure.

### 2.5. Statistical Analysis

Statistical analyzes were performed using IBM SPSS^®^ version 26 software. Continuous variables were reported as mean ± standard deviation if normally distributed or median with interquartile range (IQR) if not. Categorical variables were expressed as frequencies with percentages. Student’s *t*-test or Mann–Whitney U-test (depending on whether the values were normally distributed) allowed the comparison of continuous variables while the comparison of percentages was performed using the Pearson’s chi-squared test.

LB patients were matched 1:1 from a cohort of 417 RFA patients using nearest-neighbor matching on the linear propensity score on age (tolerance 0.10), sex (no tolerance) and LA dilatation (indexed LA volume LAVI > 34 mL/m^2^, no tolerance). A *p*-value < 0.05 was considered statistically significant.

## 3. Results

### 3.1. Baseline Characteristics

As expected with the case-control matching, LB and RFA patients had similar age, sex and LA indexed volume (Table 1). No significant difference was observed between the two groups on any of the baseline parameters, including classical predictors of AF recurrence after AF ablation, such as CHA2DS2-VASc score, left ventricular ejection fraction, obstructive sleep apnea or renal failure. In the two groups, the majority of patients had at least one antiarrhythmic drug before being referred for AF ablation. LA dilation was found in almost half of each group (44%), but the median CHA2DS2-VASc score was low (1 for each group).

### 3.2. Ablation Procedure Characteristics

Several differences were observed between the two groups with regard to the characteristics of the procedure (Table 2). Indeed, despite similar anatomical features, LB procedures were significantly shorter than RFA (129 (110–160) vs. 160 (119–198) min, *p* = 0.007), but required longer fluoroscopy durations (16.1 (12.2–24) vs. 9.8 (6.9–13.6) min, *p* < 0.001). However, the dose-area product was comparable between the two groups. LB ablation also required significantly more volume of iodinated contrast media.

Moreover, among LB procedures, we observed marked differences between the second and third-generation LBs (Table 3). Indeed, X3 procedures were associated with shorter laser applications (22 (19–29) vs. 69 (55–76) min, *p* < 0.001) and procedural durations (111 (100–128) vs. 151.5 min (128.5–167), *p* < 0.001) compared to Excalibur procedures.

### 3.3. Ablation Results

During AF ablation, two significant complications occurred in each group: one tamponade and one transient phrenic nerve palsy in the LB group; two tamponades in the RF group. Four patients required electrical cardioversion after the LB procedure (due to AF onset during catheter manipulation) vs. none in the RF group, but this difference did not reach statistical significance. No late complications were observed after hospital discharge (Table 2).

At hospital discharge, the two groups had comparable rates of beta-blockers and antiarrhythmic drug prescriptions. At the one-year follow-up (and after a blanking period of 3 months post-ablation), AF recurrence was diagnosed in 7 (14%) of the LB group vs. 14 (28%) of the RF group, *p* = 0.14. Two patients (4%) in each group had a redo procedure during the follow-up period.

## 4. Discussion

### 4.1. Main Results

The main results of our study are:

▪ LB demonstrated similar success rates at one-year follow-up than contact-force RF in paroxysmal AF ablation.
▪ Complications rates were low in each group, suggesting a satisfying safety profile for LB technology.
▪ Procedure durations were much shorter for LB, especially using the X3 balloon, than for RF, but required a longer fluoroscopic duration.

### 4.2. Efficacy

The strength of our work is to compare for the first time second and third-generation LB ablations to new generation RF ablation in paroxysmal AF.

In our study, we found similar PAF ablation success rates between laser and contact-force RF procedures, with, respectively, 86% and 72% of clinical success at the one-year follow-up (*p* = 0.14). Even if these are non-significantly different from RFA success rates, possibly due to the limited number of patients in each group, LB procedures thus appear at least as much effective as RF for PAF ablation.

These results are in line with the previous literature data: Dukkipati et al., published in 2015, the results of the only randomized trial comparing LB (first-generation) to RF ablation (without CF). They found similar efficacy for the two ablation strategies, with lower success rates than those described in our study, 61.1% and 61.7% at the one-year follow-up for LB and RF, respectively [7]. No other study has yet evaluated LB vs. RF in PAF ablation.

In the first Spanish cohort of 57 PAF patients, LB ablation was associated with an arrhythmia recurrence-free survival during a mean follow-up >1 year of 88% of patients, very close to our results [16]. Moreover, a meta-analysis on 17 published manuscripts comprising a sample of 1188 patients demonstrated a pooled estimate for 12-month freedom from atrial arrhythmia without the use of antiarrhythmic drugs for patients with PAF of 74.3% (95% confidence interval (CI), 59.9% to 86.4%) [17].

In a previously published cohort based on our center experience on 389 paroxysmal AF patients, we found a success rate of 67% at the one-year follow-up using CF RF catheters [13]. This is very close to our findings in the RF-matched cohort (72%) (sampled from this previous cohort), but lower than the rates described in the CLOSE or VISTAX studies where the reported rates were around 80% freedom of AF at 12 months [18]. This discrepancy could be explained by the AF history in our patients. In our cohort, most patients had failed at least one antiarrhythmic drug regimen before being referred for AF ablation. It is widely demonstrated that the number of antiarrhythmic drugs that failed is associated with lower success rates of ablation [19].

### 4.3. Safety

In our study, only two major complications occurred in each group (4%). One tamponade (2%) was observed in LB group, as well as a transient right phrenic palsy during right inferior pulmonary vein isolation in one of the last patients included in the cohort (which recovered a few minutes after ablation was stopped) and despite systematic phrenic nerve pacing during ablation of the right-sided PVs. In a meta-analysis published in 2018 [17], the most frequent procedural complication was phrenic nerve injury (pooled incidence 2.6%; 95% CI, 1.4% to 3.9%). However, there were only six reported cases where phrenic nerve injury persisted through the end of the study follow-up. Cardiac perforation or tamponade had a pooled estimate of 1.1% (95% CI, 0.3% to 2.3%), which is consistent with the occurrence of one case in our cohort.

In the pivotal trial on 60 patients treated with the X3 laser balloon, no phrenic injury was observed [15], compared to the 2.4% observed with the Excalibur balloon [20]. The authors suggested that this result may be explained by more antral lesion set using the more compliant balloon.

We observed 2 (4%) tamponades in the RFA group. This is slightly higher than the rate reported in large-scale studies (mostly based on electronic codes) [21]. This could be explained by the fluoroscopic guidance for transseptal puncture and/or by a selection bias of the matching procedure.

### 4.4. Procedure Duration

In our study, we found marked differences in procedure durations between LB and RF groups. Indeed, the median procedure duration was 129 min for LB vs. 160 min for RF procedures (*p* = 0.007). This is the first description of a reduced procedural time of LB compared to RF procedures. Indeed, in the trial of Dukkipati et al. on first-generation LB, LB was associated with much longer procedures than RF (236 min vs. 193 min, *p* < 0.0001). This finding is all the more interesting since RF procedure duration has significantly decreased with the use of CF and indices, such as LSI or AI [12]. In a multicenter trial comparing LB to radiofrequency in persistent AF patients, the two technologies resulted in a similar procedure duration, 135 ± 38 for LB and 128 ± 51 min for RF, *p* = 0.37 [8]. Thus, compared to the study of Schmidt et al. [8], we describe very similar results for LB procedure duration but longer RF procedures. This result highlights the larger variability in RFA procedures in terms of catheter manipulation and mapping strategies.

### 4.5. Second and Third-Generation of LB

Because of the evolution of the device during the inclusion period, 26 of the LB patients (52%) were treated with a second-generation balloon and the remaining 24 (48%) with the new X3 balloon. This allowed a head-to-head comparison between the two generations of balloons, which demonstrates shorter procedure durations with the X3, attributable to the RAPID mode. Interestingly, despite the marked shortening of the procedure, the rate of first-pass isolated PVs, complete PVI, as well that the rate of AF recurrence at one-year follow-up, was similar between the two generations of LB. In the pivotal study of the X3 balloon, the procedures were historically compared to the pivotal HeartLight study. The ablation, procedure and fluoroscopic times were significantly shorter with X3 than in the HeartLight study. PVI after the first circular lesion was achieved in 91.6% of PVs. At the one-year follow-up, patients undergoing X3 procedures had significantly higher rates of clinical success compared with the rates reported for HeartLight, (71.9% vs. 61.1%) [15].

In a further analysis focused on “single-sweep” in 100 procedures using X3 LB, the Frankfurt team achieved higher rates than ours of first-pass PVI (95% vs. 71%), probably due to their major experience in LB ablation [22].

In the context of the major increase in the number of indications for AF ablation, due to a higher level of evidence [1] and to population aging and lifestyle [23], LB shows consistent results in terms of clinical success, complications and reduced procedural duration, even in a center new to the technology, and appears an attractive alternative for PVI.

### 4.6. Limitations

Several limitations must be acknowledged. Firstly, the study design is retrospective and monocenter, and the LB procedures were performed by a single operator. However, all data from LB procedures were prospectively gathered since the beginning of the technique in the center. Secondly, our population size was relatively limited, and even if the case-control matching produced a very similar RF population compared to the LB one, the level of evidence provided by such analysis is lower than with a prospective randomized trial. Moreover, rates of treatment with an antiarrhythmic drug at one-year follow-up were not collected. Finally, while the clinical follow-up was complete for all patients, recent data suggest that closer electrocardiographic monitoring with connected devices or implantable loop recorders allow a better assessment of the success rate by measuring AF burden instead of only screening for the first recurrence episode [24].

## 5. Conclusions

In a cohort of consecutive LB paroxysmal AF ablation, both second and third-generation LBs appeared efficient and safe when compared to a matched cohort of patients having undergone RF ablation with a contact force catheter. Moreover, LB procedures were significantly shorter than RF, an important result in light of the growing rate of AF ablations in the electrophysiology laboratories. Finally, third-generation balloons demonstrated much shorter laser and procedure durations than the second-generation, thanks to the RAPID mode.

## Figures and Tables

**Table 1:** Baseline data of the overall study cohort.

Variable	Laser-Balloon N = 50	Radiofrequency N = 50	*p*-Value
Demographics			
Age—years	63 (54–68)	61 (53–65)	0.201
Male sex—no. (%)	35 (70)	35 (70)	1
BMI—kg/m^2^	26.4 (23.6–30.0)	26.2 (24.0–30.2)	0.890
Comorbidities			
Hypertension—no. (%)	18 (36)	21 (42)	0.682
Diabetes—no. (%)	3 (6)	1 (2)	0.617
Dyslipidemia—no (%)	8 (16)	19 (38)	0.023
CHA2DS2-VASc Score	1 (0–2)	1 (0–3)	0.627
Active smoking—no. (%)	4 (8)	10 (20)	0.148
Obstructive sleep apnea—no. (%)	4 (8)	6 (12)	0.739
COBP—no (%)	5 (10)	1 (2)	0.204
Obesity—no (%)	14 (28)	13 (26)	1
Stroke—no (%)	1 (2)	5 (10)	0.204
HFrEF—no. (%)	4 (8)	3 (6)	1
Coronary artery disease—no (%)	2 (4)	8 (16)	0.092
Renal failure—no (%)	3 (6)	3 (6)	1
Previous Medication			
B-blocker—no (%)	25 (50)	24 (48)	1
Amiodarone—no (%)	17 (34)	20 (40)	0.534
Flecainide—no (%)	24 (48)	20 (40)	0.420
Sotalol—no (%)	5 (10)	2 (4)	0.436
Echocardiography features			
LVEF—%	60 (55–65)	60 (56–65)	0.908
LVEF < 50%—no (%)	3 (6)	1 (2)	0.617
Left atrial dilatation—no (%)	22 (44)	22 (44)	1
Left atrial surface area—cm^2^	20 (17–23.5)	20.5 (16.9–24)	0.861
Left atrial volume indexed—mL/m^2^	33 (25–40)	32.9 (27.2–40.6)	0.532

BMI: body mass index; COBP: chronic obstructive broncho-pneumopathy; HRrEF: heart failure with reduced ejection fraction; LVEF: left ventricular ejection fraction.

**Table 2:** Atrial fibrillation ablation characteristics and follow-up.

Variable	Laser-Balloon N = 50	Radiofrequency N = 50	*p*-Value
Cardiac rhythm			
Heart rate at the beginning of procedure—bpm	60 (55–68)	60 (53–66)	0.991
AF at the beginning of procedure—no (%)	7 (14)	2 (4)	0.160
AF at the end of procedure—no (%)	5 (10)	2 (4)	0.436
Cardiac anatomy			
Left pulmonary veins—no (%)			0.480
1 (Left pulmonary trunk)	6 (12)	8 (16)	
2	44 (88)	40 (82)	
3	0	1 (2)	
Right pulmonary veins—no (%)			0.393
1 (Right pulmonary trunk)	0	1 (2)	
2	47 (94)	48 (96)	
3	2 (4)	0	
4	1 (2)	1 (2)	
Procedure characteristics			
Procedure duration—min	129 (110–160)	160 (119–198)	0.007
Iodinated contrast media—ml	2 (2–3)	2 (1–2)	<0.001
Fluroscopy duration—min	16.1 (12.2–24)	9.8 (6.9–13.6)	<0.001
Dose-area product—cGy.cm^2^	439 (250–851)	761 (334–1315)	0.324
Radiofrequency duration—min	x	29 (22.9–37.7)	
Number of RF applications—no (%)	x	52 (36–64)	
Complication—no (%)	2 (4)	2 (4)	1
Cardiac tamponade	1 (2)	2 (4)	
Transient phrenic nerve palsy	1 (2)	0	
In hospital follow-up			
Electrical cardioversion after AF ablation—no (%)	4 (8)	0 (0)	0.117
Antiarrhythmic drug at hospital discharge—no (%)	44 (88)	38 (76)	0.192
Amiodarone—no (%)	13 (26)	17 (34)	0.513
Flecainide—no (%)	27 (54)	17 (34)	0.069
Sotalol—no (%)	3 (6)	2 (4)	1
Beta-blockers at discharge—no (%)	17 (34)	12 (24)	0.271
Hospital stay duration—days	4 (4–4)	4 (4–4)	0.871
One-year follow-up			
AF recurrence—no (%)	7 (14)	14 (28)	0.14
Time to AF recurrence—days	186 (114–284)	190 (98–216)	0.860
Redo procedure—no (%)	2 (2)	2 (2)	1

AF: atrial fibrillation; RF: radiofrequency.

**Table 3:** Comparison between the second (Excalibur) and third (X3)-generation balloon procedures characteristics.

	Excalibur N = 26	X3 N = 24	*p*
Ablation			
Laser duration on left pulmonary veins—min	33 (21.5–37)	11.6 (9–17.5)	<0.001
Laser duration on right pulmonary veins—min	35 (27–42)	9 (6.6–13.5)	<0.001
Total Laser duration—min	69 (55–76)	22 (19–29)	<0.001
Number of left veins manual shots	49 (39–55.8)	8 (0–13)	<0.001
Number of right veins manual shots	51 (41–58)	0 (0–6)	<0.001
Number of manual shots	101 (89–109.8)	14 (8.3–21.3)	<0.001
Number of left veins RAPID shots	0	2.5 (0–6)	
Number of right veins RAPID shots	0	3 (0–5.8)	
Number of RAPID shots	0	12 (0–19.5)	
All PV isolated at first pass	15 (58)	17 (71)	0.388
All PV isolated at procedure end	24 (92)	22 (92)	1
AF onset during procedure	3 (12)	2 (8)	1
Procedure data			
Procedure duration—min	151.5 (128.5–167)	111 (100–128)	<0.001
Iodinated contrast media—ml	2 (2–3)	2 (2–3.8)	0.501
Fluroscopy duration—min	17.8 (12.9–23.3)	14.9 (11.5–24.4)	0.382
Dose-area product—cGy.cm^2^	443.5 (268.3–775.5)	439 (220–869)	0.645
Complication—no (%)	1 (4)	1 (4)	
Cardiac tamponade	1 (4	0	
Transient phrenic nerve palsy	0	1 (4)	
AF recurrence—no (%)	4 (15)	3 (13)	1

AF: atrial fibrillation; PV: pulmonary veins.

## Data Availability

Data are available upon reasonable request.
